# High Throughput Screening in Duchenne Muscular Dystrophy: From Drug Discovery to Functional Genomics

**DOI:** 10.3390/biology3040752

**Published:** 2014-11-14

**Authors:** Thomas J.J. Gintjee, Alvin S.H. Magh, Carmen Bertoni

**Affiliations:** Department of Neurology, David Geffen School of Medicine, University of California Los Angeles, 710 Westwood Plaza, Los Angeles, CA 90095, USA; E-Mails: tgintjee@ucla.edu (T.J.J.G.); hnocturna@ucla.edu (A.S.H.M.)

**Keywords:** dystrophin, utrophin, DMD, mdx, AONs, stop codon read-through, siRNA, RTC, Galgt2, HTS

## Abstract

Centers for the screening of biologically active compounds and genomic libraries are becoming common in the academic setting and have enabled researchers devoted to developing strategies for the treatment of diseases or interested in studying a biological phenomenon to have unprecedented access to libraries that, until few years ago, were accessible only by pharmaceutical companies. As a result, new drugs and genetic targets have now been identified for the treatment of Duchenne muscular dystrophy (DMD), the most prominent of the neuromuscular disorders affecting children. Although the work is still at an early stage, the results obtained to date are encouraging and demonstrate the importance that these centers may have in advancing therapeutic strategies for DMD as well as other diseases. This review will provide a summary of the status and progress made toward the development of a cure for this disorder and implementing high-throughput screening (HTS) technologies as the main source of discovery. As more academic institutions are gaining access to HTS as a valuable discovery tool, the identification of new biologically active molecules is likely to grow larger. In addition, the presence in the academic setting of experts in different aspects of the disease will offer the opportunity to develop novel assays capable of identifying new targets to be pursued as potential therapeutic options. These assays will represent an excellent source to be used by pharmaceutical companies for the screening of larger libraries providing the opportunity to establish strong collaborations between the private and academic sectors and maximizing the chances of bringing into the clinic new drugs for the treatment of DMD.

## 1. Introduction

Muscular dystrophies are a group of heterogeneous diseases characterized by progressive muscle wasting that ultimately lead to wheelchair dependency and premature death. They have been largely classified based on clinical presentation, pattern of inheritance, age of onset, and overall disease progression. Among those, Duchenne muscular dystrophy (DMD) is the most prominent and one of the most severe. DMD is an X-linked recessive disorder affecting 1 in 3,500 to 1 in 5,000 males and is caused by genetic defects in dystrophin, one of the largest genes identified to date [1,2,3]. The gene encompasses approximately 2.5 megabases of genome encoding 79 exons that, in skeletal cardiac and smooth muscles, result in the expression of a 427 kilodalton (kDa) protein. Mutations in the dystrophin gene account primarily for large deletions or single point mutations and, to a lesser extent, for insertions, duplications and translocations, all leading to the disruption of the coding reading frame of the dystrophin mRNA and resulting in absence of dystrophin expression throughout the body. In addition to DMD, a much milder form of muscular dystrophy, known as Becker muscular dystrophy (BMD), has been reported. This dystrophinopathy is generally characterized by large deletions of the dystrophin gene that do not alter the coding reading frame of the mRNA and that therefore, results in the expression of a shorter although partially functional dystrophin protein. Phenotypically, BMD patients present with a wide spectrum of symptoms and the pathophysiology and prognosis are generally less severe than that of DMD with some cases reported to be asymptomatic until late adulthood.

**Figure 1:**
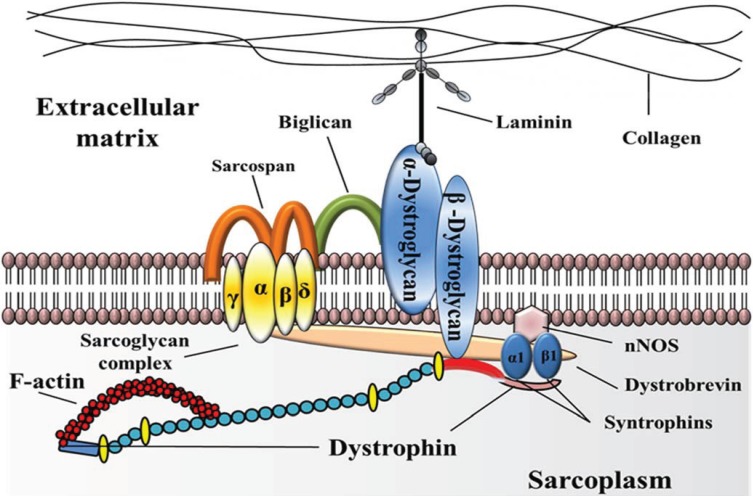
Structural organization of the dystrophin protein and its interacting partners. Dystrophin is positioned underneath the basal lamina and extends through the sarcoplasm, serving as a critical component of the dystrophin-associated protein complex (DAPC). The dystrophin protein binds cytoskeletal F-actin through its N-terminus domain and the DAPC through its C-terminus, acting as an important link between the internal cytoskeleton and the extracellular matrix. The central rod domain is formed by triple-helical segments similar to the repeat domains of spectrin which are interrupted by four hinge regions. The C-terminal region binds β-dystroglycan as well as the syntrophins and α-dystrobrevin. In addition, dystrobrevin links dystrophin with the sarcoglycan-sarcospan complex which is also indirectly linked to dystrophin through the dystroglycan complex (α-dystroglycan and β-dystroglycan). Redrawn from Fairclough *et al*. and Rahimov *et al*. [56,57].

While the function of the dystrophin gene has not been completely elucidated, the large structure and complexity of the protein suggests that one of its roles is to provide structural integrity to skeletal and cardiac muscles by linking the subsarcolemmal cytoskeleton to the extracellular matrix through the dystrophin-associated protein complex (DAPC) (Figure 1). In DMD, the absence of dystrophin leads to a drastic reduction of components of the DAPC from the sarcolemma which ultimately causes an increased susceptibility to muscle damage in response to physical activity or injury and increased necrosis of myofibers [4]. The dysregulation of calcium ions, calcium channels and calcium signaling pathways seems to further exacerbate the pathophysiology of the disease [5].

Corticosteroids have been shown to slow the progression of the disease and to prolong the lifespan of the patients, but their use is associated with strong side effects and their exact mechanisms of action is not entirely clear [6,7,8]. Years of research have enabled the development of new approaches some of which have already entered clinical trials with promising results. Viral vectors have been successfully engineered to accommodate shorter, although still functional dystrophin genes capable of compensating for the lack of dystrophin [9,10,11,12,13,14,15,16,17]. Similarly, the use of antisense oligonucleotides complementary to regions of the dystrophin premature mRNA have been successfully used to induce skipping of one or more exons to produce in frame, although shorter, dystrophin proteins [18,19,20,21,22,23]. Although effective, these approaches can only promise to convert a severe DMD phenotype into a BMD and, as such, they are not ideal. As a result, efforts have been made toward the identification of strategies capable of restoring full-length dystrophin. Among those, read-through of premature stop codons [24,25,26,27,28,29] and gene editing mediated by single-stranded oligonucleotides [30,31,32] or, more recently, nucleases [33,34,35] have gained attention. Alternative strategies to DMD have focused on upregulating the expression of proteins like utrophin that could compensate for the loss of dystrophin [36,37,38,39,40,41] or have aimed at ameliorating the pathology by increasing muscle strength [42,43,44], reducing muscle fibrosis [45,46,47], and decreasing inflammation [48,49,50,51,52,53]. Additional pharmacological approaches have also been pursued or are currently under development that target pathways known to be altered in DMD as a result of the lack of dystrophin as reviewed elsewhere [54,55]. Despite the advances that have been made, there is currently no cure for the disease and treatments can only manage the symptoms.

High throughput screening (HTS) has recently emerged as a powerful tool in identifying new active compounds that could be used to treat the disorder as well as to discern mechanisms underlying certain pathological conditions. Furthermore, the development of core facilities in academic settings capable of screening thousands of molecules within any given assay has enabled many investigators interested in understanding the pathophysiology of DMD to develop assays to study a number of biological processes. Most HTS platforms have used simple assays based, primarily, on the transient or stable expression in eukaryotic cells of a single reporter vector. In these systems, expression of the vector from cells is detected through enzymatic activity, such as in the case of assays that use a luciferase reporter, or through high content imaging as in the case of reporters expressing a fluorescent protein like the Green Fluorescent Protein (GFP). Changes in expression of these reporters are then used as major readout. The widespread use of these simple assays is primarily due to the ease by which these vectors can be produced and the relatively low cost associated with the screening of libraries. However, few screens have recently been developed that rely on more refined assays. These assays have utilized cell lines isolated from transgenic models overexpressing more than one reporter gene thus allowing to better control for false positives. The use of dual reporter systems can also be used to discriminate between different signaling pathway being activated in response to treatment with compounds or other active molecules such as small interfering RNA (siRNA) or complementary DNA (cDNA). Others HTS platforms have implemented the use of specific antibodies directed toward the protein being expressed to increase the sensitivity and robustness of the screen.

This review will highlight some of the studies that have been described over the past few years and that have employed HTS to identify new molecules that could be used to treat DMD or those that have implemented the technology to better understand the biological functions that control muscle regeneration and muscle repair and their current stages of development in the clinical arena.

## 2. HTS of Compounds Capable of Suppressing Nonsense Mutations

Nonsense mutations are relatively common among DMD accounting for approximately 13% of the genetic defects detected in patients [58]. These premature termination codons (PTCs) are generated by single base substitutions that result in stop codons (TAG, TAA and TGA) and that lead to the expression of a nonfunctional, truncated dystrophin protein.

The ability of antibiotics, such as streptomycin, to induce misreading of the mRNA has been known for almost three decades [59,60]. However, the possibility of using specific antibiotics like gentamycin to precisely suppress nonsense mutations has emerged as a therapeutic approach only during the last 20 years or so [24,61,62,63]. The mechanisms of action of these read-through compounds (RTCs) are not well understood, but several lines of evidence suggest that these small molecules act by interfering with normal translation and proofreading abilities of ribosomes allowing transcription to continue and to produce a functional protein [64]. Clinical trials in DMD patients using gentamicin have been disappointing with limited or no beneficial effects achieved in patients [25,26,27]. Furthermore, the use of gentamicin is associated with strong side effects, limiting long term administration of this drug and restricting its clinical use. Under these circumstances, the use of HTS to identify compounds capable of promoting nonsense suppression has proven to be a valuable tool for DMD [28,65].

To date, three major screens have been reported and each has led to the identification of new RTCs. The first screen was performed by PTC Therapeutics, a biopharmaceutical company focused primarily on the discovery and development of small-molecules that target post-transcriptional control processes. The platform implemented by the company utilized a luciferase reporter construct (LUC-190) containing a TGA nonsense codon in the middle of the luciferase coding region. The PTC presence in the construct reduces the expression of functional luciferase proteins unless incubated with a compound capable of inducing read-through of the nonsense mutation. The assay was used to screen approximately 800,000 low molecular weight compounds that were tested using two independent assays (Table 1). The first HTS assay employed human embryonic kidney (HEK293) cells stably transfected with the LUC-190 construct and analyzed for their ability to induce luciferase activity 16 h after exposure to compounds [28]. The second HTS used a cell-free translation system in which synthetic LUC-190 mRNA and rabbit reticulocyte lysate were incubated for 2 h with the library compounds. These screens have enabled the identification of PTC124 (or Translarna, formerly known as Ataluren), a nonaminoglycoside RTC that has been further brought into clinical development for the treatment of DMD and cystic fibrosis. PTC124 has been shown not to affect read-through of normal termination codons and to have limited to no off-target effects *in vitro* as determined by analyses at the mRNA and protein levels.

However, not long after the publication of the study describing the assay, reports have demonstrated that one of the properties of PTC124 is to stabilize the luciferase protein, which results in an increase of luciferase activity in the presence of the compound that is independent of its ability to suppress nonsense mutations [66,67]. This stabilizing ability is likely to have led to an overestimation of the efficacy of the compound to read-through PTCs during the initial stages of selection of primary hits thus affecting all subsequent stages of lead optimization. While a possible explanation to this has been offered [68], further studies have collected substantial evidence of PTC124’s off-target effects [69]. Importantly, the apparent lack of specificity of the luciferase-based assay used to identify PTC124 has served as a warning to the industrial and academic sectors of possible bias effects of HTS platforms implementing reporter systems and has helped establish some of the parameter and quality controls necessary when designing HTS systems utilized by industry and academia for the identification of compounds.

Despite the controversies surrounding this molecule, PTC124 has, in fact, been shown to promote nonsense suppression and to partially restore expression of cystic fibrosis transmembrane conductance regulator (CFTR) in cystic fibrosis animal models as well as dystrophin in *mdx* mice, a widely used animal model for DMD. The model is characterized by a nonsense mutation in exon 23 of the dystrophin gene that leads to absence of dystrophin expression in skeletal and cardiac muscles [28,29,70,71,72,73,74]. Clinical trials using PTC124 have shown promising results during phase I and phase IIa studies, but have failed to meet primary endpoints in phase IIb studies conducted in DMD patients. In fact, results from the phase IIb trial demonstrated only limited activity in the ability of patients to preserve muscle function over the course of 48 weeks of treatment, raising questions over PTC124’s efficacy for the treatment of this disease [75]. Currently, PTC124 is being evaluated in phase III studies for DMD and the results are expected to be released within a year or so.

**Table 1:** Therapeutic drugs to DMD identified through HTS.

Approach and Lead Candidate/s	Size of Library	Reporter or Assay	Screening System	Animal Model Used to Validate Hits *In-Vivo*	References
**Read-Through of Premature Stop Codons**					
PTC124 (Translarna)	800,000 Compounds	Luciferase Reporter	Human HEK 293 Cells	*Mdx* Mice	[28]
RTC13	35,000 Compounds	PTT-ELISA Assay	Rabbit Reticulocytes	*Mdx* Mice	[65,76,77]
**Compounds that Enhance AON Activity**					
Podophyllotoxin and HDAC inhibitors	10,000 Compounds	Luciferase Reporter	Human HEK 293 Cells	None	[78]
6-thioguanine (6TG)	2,000 Compounds	GFP Reporter	Murine C2C12 Mbs	*Mdx* Mice	[79]
Dantrolene	300 Compounds	GFP Reporter	Murine C2C12 Mbs	*Mdx* Mice	[80]
**Utrophin Upregulation**					
SMTC1100	Not Specified	Luciferase Reporter	Murine H2K Cells	*Mdx* Mice	[41,81,82]
Nabumetone	1,124 Compounds	Luciferase Reporter	Murine C2C12 Mbs	None	[83]
**Compounds that Alter Glycosylation**					
Lobeline	1,124 Compounds	Chemiluminescent Assay	Murine C2C12 Mbs	None	[84]
**Inhibitors of TGF-β Signaling**					
Several hundred hits	2,500 Compounds	Luciferase Reporter	Human HEK 293 Cells	None	[85]
**PDE5 Inhibitors**					
Aminophylline and Sinedafil	2,640 Compounds	Survival Assay	Zebrafish *sapjie* and *sapjie*-like	*Mdx^5cv^* Mice	[86,87,88]
**Regulators of Cell Differentiation and Lineage Commitment**					
Geraldol and Bromopride	1,600 Compounds	Morphological Assays	Human Muscle Cells	None	[89]
miR-1.2, miR-133, and miR-206	875 miRs	GFP-Reporter	Human HEK293 Cells	*Mdx* Mice	[90]
Forskolin	2,400 Compounds	Dual GFP/mCherry Reporters	Zebrafish Blastomers	NOD/SCID/IL2Rγ^−/−^ and *Mdx* Mice	[91]

A different system, completely devoid of luciferase reporter assays, was described by Du *et al*. in 2009 [65]. The screening platform utilizes a protein transcription/translation (PTT) assay based on the detection of full-length protein expression resulting from the transcription and translation of a plasmid containing a nonsense mutation in the ATM gene. In this assay, protein expression was detected through enzyme-linked immunosorbent assay (ELISA) which allowed to specifically detect only full-length ATM protein and to accurately quantitate the amount of protein being produced in the presence of the RTC [65]. This PTT-ELISA assay was used to initially screen approximately 34,000 compounds and has led to the identification of RTC13. The ability of this newly identified compound to suppress nonsense mutations in the dystrophin gene was assessed in *in vitro* and *in vivo* in *mdx* mice. Systemic administration of RTC13 showed to be able to restore dystrophin expression in virtually every muscle analyzed including diaphragm and heart, two of the muscles that are most affected in DMD patients and that are proven to be the most difficult to target by the majority of the therapeutic approaches currently under development. Furthermore, the levels of dystrophin protein restored in muscle was significantly higher than those achieved by PTC124, demonstrating the clinical relevance that this compound may have for the treatment of DMD [29]. At the moment, RTC13 remains in the preclinical stages of development and, if proven to be safe to be tested in patients, is expected to enter clinical testing within the next few years.

A third screen implementing the same PTT-ELISA assay used to identify RTC13 has recently been described by Du and colleagues [77]. The screen which was performed on an additional 30,000 compounds has led to the identification of potential new candidate drugs with read-through activity. The ability of these new compounds to suppress nonsense mutations in cells or animal models for DMD has not been validated yet. However, it is possible that, if not all, at least some of those new RTCs may become a valid alternative to RCT13 or PTC124 thus expanding the number of compounds available for the treatment of DMD due to nonsense mutations.

## 3. HTS of Small Molecules that Enhance Skipping of the Dystrophin Gene

Among the treatments currently being investigated for DMD, antisense-mediated exon skipping is one of the most promising. The approach employs small antisense oligonucleotides (AONs) complementary to regions of the dystrophin pre-mRNA and designed to anneal to regulatory elements that control splicing of intron/exon sequences during the assembly of mature dystrophin mRNA transcripts. Annealing of AONs to these regions has been shown to induce skipping of one or more exons and to restore the expression of in-frame transcripts, which can then be translated into shorter, although still functional, dystrophin proteins similar in structure to those detected in BMD patients [18,20,92,93,94].

Current clinical trials using AONs have focused on targeting and induce skipping of exon 51 of the dystrophin pre-mRNA and have used either PRO051 (also known as GSK2402968 or Drisapersen), an AON made of 2ꞌ-*O*-methyl phosphorothioate bases (2ꞌOMePS) or AVI4658 (also known as Eteplirsen), an antisense oligomer made of morpholino nucleic acid [95,96]. This particular exon has been chosen based on hierarchical analyses of the different types of deletions of the dystrophin gene reported to date in patients and by prioritizing the selection of specific exons that, when skipped, could restore the coding reading frame of the dystrophin mRNA in the largest possible number of patients. AON-mediated skipping of exon 51 is believed to be applicable to the larger majority of the DMD patients characterized by deletions of the dystrophin gene and could potentially treat 13% of Duchenne boys.

**Figure 2:**
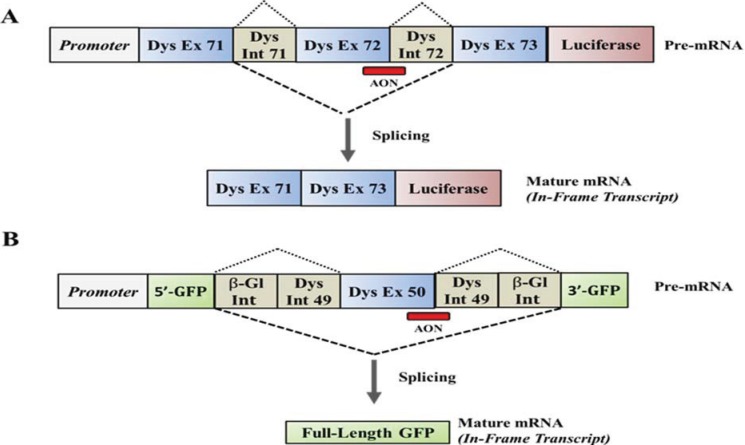
Schematic representation of the reporter constructs used to identify compounds or genes that influence the ability of AON to enhance skipping of the dystrophin gene. (**A**) The hE72-Luc DMD minigene construct used by O’Leary *et al*. [78] contains the region of the dystrophin gene spanning from the 5ꞌ-end of exon 71 through the 3ꞌ-end of exon 73. The luciferase coding sequence was inserted downstream the hDMD minigene. The resulting mRNA transcript is out of frame resulting in absence of luciferase expression. Annealing of an AON targeting the exon/intron junction of exon 72 induces skipping of the exon and corrects the coding frame of the luciferase mRNA inducing the expression of a protein containing the region of the dystrophin gene encoded by human exons 71 and 72 linked at it 3ꞌ region to full-length luciferase; (**B**) The reporter vector used by Hu *et al*. [79] and Kendall *et al*. [80] contains the 5ꞌ region of the GFP cDNA upstream a minigene consisting in a portion of the human β-globin intron followed by the intron/exon flanking regions of the human dystrophin exon 50 and the remaining region of the human β-globin intron. The 3ꞌ-region of the GFP gene was then cloned downstream of the minigene. Skipping of exon 50 mediated by an AON designed to anneal to the acceptor site of human exon 50, eliminates the minigene and allows the expression of full-length GFP; Dotted lines represent splicing of intronic sequences from the pre-mRNA while dashed lines represent those achieved in the presence of AONs designed to skip the exon responsible for the lack of dystrophin.

Although the results of the clinical trials are promising [97,98], the levels of dystrophin expression achieved in animal models and in DMD patients are not optimal. As a result, drugs that can increase the ability of AONs to interact with the region of the dystrophin pre-mRNA targeted for correction and increase the frequencies of exon skipping achieved in muscle are likely to have important therapeutic implications for the treatment of the disease. Subsequently, over the past few years there has been increasing interest from different groups within industry and academia to identify compounds that could be co-administered systemically with AONs to enhance exon skipping. All the screens performed to date have utilized a reporter system in which intronic and exonic regions of the dystrophin gene were cloned upstream or within the coding sequence of a luciferase or a GFP cDNA. The presence of an AON designed to anneal and induce the skipping of the exon responsible for producing an out-of-frame transcript would restore the coding reading frame of the reporter gene thus allowing the expression and detection of the protein (Figure 2).

The Novartis Research Foundation has been the first to report the identification of several active compounds capable of enhancing AON-mediated exon skipping of a DMD minigene construct. The construct utilized for the screens contained the region of the dystrophin gene spanning from the 5ꞌ-end of exon 71 to the 3ꞌ-end of exon 73 immediately upstream of the luciferase coding sequence (hE72-Luc). Skipping of exon 72 results in the restoration of the coding frame of the luciferase mRNA and the expression of the region of the dystrophin protein encoded by exons 71 and 73 linked to the N-terminal region of luciferase (Figure 2A) [78]. As a positive control, they utilized Trichostatin A (TSA) which was selected after screening a panel of histone deacetylase (HDAC) inhibitors known to enhance transcription and splicing [99,100]. Screens were conducted in the presence or absence of a 2ꞌOMePS AON designed to skip exon 73 and were performed using approximately 10,000 known small molecules derived from public databases. Secondary and tertiary screenings demonstrated that many of the 21 unique structures identified were able to induce skipping of the dystrophin gene in *mdx* myotubes even in the absence of AONs suggesting lack of specificity of those compounds. The most potent small molecules identified were podophyllotoxin tubulin modulators and two HDAC inhibitors: TSA and Scriptaid. In an effort to gather further insights into the potential mechanisms of action of the newly identified compounds and in order to identify new genetic targets that play active roles during exon skipping in the presence or absence of AONs, the group performed a genome-wide cDNA overexpression screen of approximately 17,000 cDNA clones and identified several genes involved in RNA stability, RNA processing, chromatin modification and genes involved in cell-cycle progression [78]. The possible involvement of genes that control cell-cycle regulation was further validated through a siRNA screen targeting and inhibiting the expression of all known protein kinases. Despite the encouraging results obtained, the newly identified compounds have not yet advanced into preclinical and clinical testing and the actual state of the development of antisense drugs that enhance AON-mediated exon skipping of the dystrophin gene by Novartis remains unknown.

Two additional screens aimed at identifying drugs that enhance dystrophin exon skipping have been described [79,80]. The screens conducted by Hu and colleagues [79] and that performed by Kendall *et al*. [80] have implemented the same reporter construct generated in the laboratory of Qi-Long Lu in collaboration with Ryszard Kole [79,101]. The construct contains the intron/exon flanking regions of dystrophin exon 50 juxtaposed by a sequence of the human β-globin intron at its 3ꞌ- and 5ꞌ- regions. The genetic sequence was then inserted within the central coding sequence of GFP to generate a construct in which splicing and inclusion of exon 50 in the coding reading frame of GFP will result in an out-of-frame transcripts leading to lack of GFP expression [79,101]. The presence of an AON designed to target splicing regulatory elements that control the inclusion of exon 50 into the mature mRNA allows the expression of GFP (Figure 2B) [79]. The reporter construct was used to transfect C2C12 myoblasts and to screen 2,000 bioactive compounds contained in the Spectrum collection [102] for the screen described by Hu *et al*. [79] or 300 small molecules present in the BioMol Library [103] for the HTS performed by Kendall and collegues [80] (Table 1). Screening of the Spectrum collection of compounds has led to the identification of 6-thioguanine (6TG), an antimetabolite used in chemotherapy for cancer treatment. The compound is a purine analogue of the nucleobase guanine that has been shown to be incorporated into DNA and to interact with DNA structures, therefore altering the structure and stability of DNA duplexes. Although the mechanisms of action of 6TG have not been explored in detail, results obtained in cells in culture and *in vivo* in *mdx* mice suggests that the activity of the compound in enhancing skipping may be independent from the presence of the AON, raising questions on the feasibility of using the molecule in patients. Recent studies have also questioned the feasibility of using 6TG to enhance AON activity due, primarily, to a lack of specificity for regions of the dystrophin pre-mRNA other than those targeted for skipping and the limited effects achieved *in vivo* following administration in *mdx* mice [104].

The screening performed by Kendall and colleagues using the BioMol library has led to the identification of a number of active compounds [80]. Among those, Dantrolene, a drug approved by the Food and Drug Administration (FDA), was selected as the lead compound despite not having the highest efficacy among the molecules identified, due to clinical studies conducted on DMD patients and animals models for DMD that showed modest, although detectable, improvements in muscle pathology [105,106,107]. Dantrolene is a muscle relaxant known to inhibit excitation–contraction coupling in muscle, likely through its binding to the ryanodine receptor (RyR1) and by preventing the release of calcium from the sarcoplasmic reticulum. Changes in calcium homeostasis is the primary mechanism that controls contraction of skeletal muscle and has also been implicated in muscular dystrophy as one of the causes that leads to degradation and apoptosis of muscle fibers [108,109]. Several reports have also demonstrated that restoring calcium homeostasis may lead to improvement in muscle function and muscle strength [110,111,112]. The study by Kendall *et al*. eloquently demonstrated that systemic administration of Dantrolene can specifically enhance the activity of AONs designed to skip the exon responsible for the lack of dystrophin in *mdx* mice following intramuscular or systemic administration of the oligonucleotide. The levels of dystrophin expression detected in mice that received Dantrolene in conjunction with the AON were up to three-fold higher than those detected in mice that received oligonucleotides alone. Similar results were obtained in muscle cells isolated from a DMD patients characterized by a deletion of the dystrophin gene comprised between exons 45 and 50 treated with an AON designed to skip exon 51 of the dystrophin mRNA and capable of restoring the coding reading frame of the transcript, further supporting the clinical relevance of Dantrolene for the treatment of the disease. The exact mechanism that enables Dantrolene to exert its activity is not clear, but it appears to require the presence of the AON as treatment of cells or *mdx* mice with the drug alone leads to little or no detectable skipping of dystrophin mRNA. Additional studies are likely to be required before Dantrolene can be tested in clinical trials. However, the fact that this drug has already been tested in DMD patients suggests that its combinatory use with AONs could move into clinical testing in a relatively short period of time. Intriguingly, the possibility that the same drug could act not only by promoting exon skipping, but also by restoring calcium homeostasis in muscle also suggests that its use in the clinic may be preferable over other drugs that can only promote AON activity.

## 4. Upregulation of Utrophin for the Treatment of DMD

**Figure 3:**
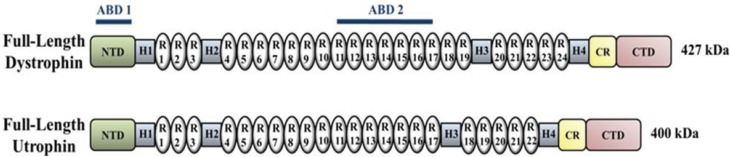
Structural domains of the dystrophin and utrophin proteins. The dystrophin and the utrophin proteins share a similar organization and binding affinity for other members of the DAPC. Both proteins contain an N-terminal domain (NTD) that interacts with actin, also known as actin-binding domain 1 (ABD1), a central region composed of different spectrin-like repeats (R1–R24) interspersed by four hinge regions (H1–H4), a cysteine-rich (CR) domain that binds β-dystroglycan and a C-terminal domain (CTD) that interacts with β-dystrobrevin and the syntrophins. The major difference between the two proteins is represented by the presence of a second actin-binding domain (ABD2) present in dystrophin, but absent in utrophin.

Although restoration of dystrophin is considered to be the ideal option for the treatment of DMD, concerns have been raised over the possibility that the levels of protein expression currently achieved by most of the therapeutic approaches under development may not be sufficient to confer muscle stability. Even if those levels reach clinically relevant amounts, concerns still remain over the possibility that the protein may be recognized as an antigen therefore eliciting an immunological response. Consequently, efforts have concentrated on identifying alternative approaches that could be used to substitute for, or in combination with, those aimed at restoring dystrophin in muscle. Among these, upregulation of utrophin is one of the most promising. Utrophin is a dystrophin-related protein whose expression has been shown to partially compensate for the absence of dystrophin and to help rescue the phenotype in animal models for DMD [37]. Dystrophin and utrophin are highly homologous and both proteins show similar structure and organization of the different domains (Figure 3) [113,114,115]. Importantly, upregulation of utrophin is thought to be safe in patients due to the fact that the protein is constitutively expressed in different organs and tissues, including muscle, and therefore should not be immunogenic, although no evidence has been provided yet in support of such a claim. Notably, a drug capable of upregulating utrophin at the levels required to achieve beneficial effects in DMD boys could potentially be applicable to all patients irrespective of their mutations on the dystrophin gene [41], a trait that makes this approach a particularly valuable option for the treatment of DMD.

Like dystrophin, the utrophin gene is quite large, occupying approximately 900 kb of chromosome 6 and encoding for a protein of approximately 400 kDa. Its expression is controlled by two different promoters (promoter A and promoter B). The A promoter controls the expression of the gene in skeletal muscle, while the B promoter is active in the heart, lung and endothelial cells. Several studies aimed at better characterizing the utrophin A promoter have demonstrated that its transcriptional activation is, at least in part, regulated by heregulin (HRG), a molecule capable of activating the ETS-related transcription factor complex GA-binding protein (GABP) composed by the α and β subunits (GABPα/β) whose role appears to be critical in initiating utrophin gene expression in muscle cells in culture [116,117]. This hypothesis is supported by *in vivo* studies that demonstrated that intraperitoneal injections of a small peptide encoding the epidermal growth factor-like region of HRG ectodomain in *mdx* mice results in up-regulation of utrophin. The increase in utrophin expression resulting from the administration of the peptide is sufficient to improve muscle function and to reduce muscle disease pathology [40]. Several other studies have confirmed the possibility of increasing expression of utrophin by targeting specific transcription factors in the utrophin A promoter, further demonstrating the validity of this approach for the treatment of DMD [118,119,120,121,122]. However, the use of HTS to identify molecules that could upregulate utrophin expression by acting directly on the A promoter, has only emerged as a potential application to DMD during the past few years [123].

Four studies were described in 2011, three of which utilized dystrophin-null H2K murine cell lines stably expressing a reporter vector generated by cloning the human utrophin promoter and its first untranslated exon upstream the luciferase gene (utrnA-luc) [41,81,82]. Increases in luciferase expression were then quantified at both, the mRNA and protein levels. These studies have culminated in the selection and optimization of an orally bioavailable compound named SMTC1100.

Daily administration of SMTC1100 in *mdx* mice has been shown to induce statistically significant increases in the levels of utrophin in muscles, confirming its function *in vivo*. The increase in utrophin expression detected in mice has supported the feasibility of using SMTC1100 for the treatment of DMD and has provided clues on the levels of utrophin expression needed in patients to achieve functional effects. However, Phase I trials sponsored by BioMarin Pharmaceutical Inc. and Summit Corp. plc have failed to demonstrate acceptable plasma levels in subjects, leading BioMarin to discontinue its involvement in further developing this molecule for DMD. Eventually, a second phase I trial using a more bioavailable formulation of SMTC1100 has successfully proven the desired safety profile of the compound in human volunteers while demonstrating the ability of the molecule to efficiently achieve the desired plasma levels expected to be efficacious in patients [124,125]. These safety and feasibility studies have paved the way for the ongoing phase Ib study in DMD patients. Although the full report on study results and outcomes of the clinical trial will have to await further analyses, preliminary results suggest that SMTC1100 is safe and well tolerated in patients and that the majority of DMD boys that received the compound showed reduced creatine kinase levels following dosing, suggesting some efficacy of the compound.

The fourth study was conducted by Moorwood *et al*. in C2C12 muscle cells expressing a reporter vector containing a 2.3 kb human utrophin A promoter fragment inserted upstream to the coding sequence of the luciferase gene. The system was used to screen the Prestwick library, a collection of approved drugs and a library containing natural compounds [83]. The screen has led to the identification of Nabumetone, a non-steroidal anti-inflammatory drug that inhibits the cyclooxygenase enzyme and is commonly used to reduce inflammation and pain in patients with arthritis [83]. The drug was shown to increase endogenous utrophin mRNA by 80% and endogenous utrophin protein by 20% with limited or no toxicity detected in culture. Although interesting, the study was limited to demonstrate of the feasibility of using the assay to identify new compounds and no data are currently available on the efficacy of the compound *in vivo* or the current status of development of this drug as a potential upregulator of utrophin for the treatment of DMD.

More recently, a new assay has been developed and validated in muscle cells which utilizes a reporter vector in which a luciferase coding sequence is flanked by the utrophin 5'- and 3'-untranslated regions (UTRs) [126]. This system may become a valuable tool for the identification of additional compounds capable of upregulating utrophin expression in skeletal muscle. Notably, assays similar to those developed thus far could be used to identify small molecules or compounds that target the B promoter of the utrophin gene thus expanding the applicability of HTS to the discovery of drugs capable of increasing utrophin expression into cardiac muscle.

## 5. Screening of Compounds that Alter Glycosylation of Members of the Dgc

The work of Martin and colleagues has clearly demonstrated that altering glycosylation of α- or β-dystroglycan (Figure 1) has profound effects on the ability of muscle cells to bind dystrophin and that overexpression of cytotoxic T cell (CT) N-acetylgalactosamine (GalNAc) transferase (Galgt2) in skeletal muscle of *mdx* mice can promote the association of utrophin with the glycoprotein complex [121,122,123,124,125]. Furthermore, overexpression of the Galgt2 enzyme has been shown to increase expression of other components of the dystroglycan complex, including α- and β- dystroglycan, as well as α-, β-, and γ-sarcoglycans (Figure 1) whose expression is affected in dystrophic muscles as a result of the lack of dystrophin.

Based on these observations, Cabrera *et al*. have developed an HTS capable of recognizing and identifying compounds that alter glycosylation [84]. The screen utilizes the plant *Wisteria floribunda* agglutinin (WFA), a lectin that appears to preferentially bind carbohydrate structures terminating in N-acetylgalactosamine linked at specific positions in the galactose and preferentially recognizes the GalNAc [127]. In the screen, C2C12 cells were incubated with compounds present in the Prestwick library and binding of WFA to myotube membrane was measured using a biotin-streptavidin chemiluminescent-based assay. Of the six compounds identified, Lobeline, a drug that is FDA-approved as an aid for smoking cessation, was selected as lead compound due to its ability to increase WFA binding up to four-fold in C2C12 myotubes exposed to the drug compared to untreated cells [84]. Although interesting, the study did not examine the effects of Lobeline in *mdx* mice and as such, the potential of this drug for the treatment of DMD still remains largely confined to proof-of-concept studies *in vitro*.

## 6. HTS in Regenerative Approaches for DMD

While HTS of small molecules has proven to be a valid approach in the discovery of new drugs that could treat DMD, its use in other disciplines, including basic sciences, has already shown its potential for the discovery of new processes and mechanisms that regulate muscle function. Within the last two years alone, several screens have been described aimed at identifying compounds or genes that influence the activation and differentiation of muscle stem cells and muscle progenitor cells as a potential approach to enhance the ability of these cells to regenerate diseased muscle.

One of the hallmarks that characterize DMD patients as well as other muscle diseases, such as aging and sarcopenia, is the inability of muscle stem cells known as satellite cells (SCs) to efficiently regenerate muscle as a result of the degenerative process that occurs in diseased tissue or as the result of injury. SCs are located beneath the basal lamina of muscle fibers and are the primary source of stem cells responsible for the homeostasis and repair of skeletal muscle. Under normal conditions, they are quiescent, but are rapidly activated in response to damage or injury. Activation leads to replicative expansion and the production of daughter cells that will either enter the myogenic lineage progression to become myoblasts or withdraw from the cell cycle to reenter the quiescent stage [128,129,130]. SCs can be easily dissociated from muscle fibers and, when placed in culture, they are known to rapidly activate and divide. However, to this date, SCs cannot be efficiently maintained in their undifferentiated state and, once explanted and expanded in culture, they are known to rapidly become myoblasts. As a result, most of the HTS that have been performed to date and that have aimed at identifying compounds or genes that influence regeneration and repair of skeletal muscle have used either C2C12 or primary myoblast cell lines isolated from human or mouse muscles and expanded in culture [85,89,90,131].

The first HTS system to be reported was described by Cash *et al*. [85] and was aimed at developing a potential platform that could efficiently identify compounds that inhibit myostatin, a member of the transforming growth factor (TGF)-β family of secreted ligands known to be a strong negative regulator of muscle growth [132,133,134,135,136].Together with activin A, another member of the TGF-β family also known to regulate muscle growth, myostatin bind type II activin receptors and signal through the TGF-β signaling pathway involving SMAD phosphorylation. Myostatin is thought to bind with greater affinity to the type II B receptor (ActRIIB) and activin A to the type IIA activin receptor (ActRIIA). To date, therapeutic approaches to DMD and other muscle disorders characterized by loss of muscle mass that have reached testing phases in humans have focused primarily at inhibiting myostatin or activin-binding proteins such as follisatin to promote regeneration mediated by SCs. So far, a recombinant human antibody named Stamulumab (MYO-029), which was designed to bind and inhibit myostatin, has shown only moderate effects in Phase I and II clinical trials in patients with BMD, limb-girdle and fascioscapulohumeral muscular dystrophy [137]. Similarly, the activin blocker ACE-031, although showed an improvement in DMD boys, appears to be poorly tolerated in patients. A second myostatin blocker, called PF-06252616, is currently in Phase I clinical trials in healthy volunteers and it may enter clinical testing in patients within the next year or so. Given the relatively low number of treatments available, the focus of many researchers and pharmaceutical companies has now centered on the identification of new molecules and new pathways that could be used or targeted to enhance muscle regeneration in an effort to counteract the muscle loss typical of DMD.

Cash *et al*. used HEK293 cells stably expressing a luciferase reporter construct and exposed to either myostatin or activin A in order to stimulate TGF-β signaling pathway [138,139,140,141]. The screening of 2,500 compounds led to the identification of over 700 compounds as potential inhibitors of myostatin or activin A. Importantly, several of the compounds identified appeared to specifically inhibit myostatin while leaving unaltered the inhibition of activin A [85]. Although the results are still preliminary, they demonstrate the feasibility of developing HTS assays specifically designed to discriminate between ligands of the TGF-β signaling pathway and, as such, this assay may become an important tool in the identification of compounds and drugs that could be moved into clinical testing in the near future.

A second HTS, described by Khanjyan *et al*., was specifically developed to identify genes that, like myostatin, are implicated in muscle regeneration and growth [131]. The screen is unique because it is the first one to utilize libraries of siRNAs rather than compounds, making this the first functional genomic HTS to have been developed and implemented in the study of myogenesis. The screen has enabled the identification of several known and unknown biological functions linked to pathways that are critical to terminal differentiation of myoblasts, including cell survival, growth and differentiation as well as signaling pathways that take an active role during myogenesis. *In vivo* analyses using transplantation of myoblasts downregulating the primary hits identified by the screen and performed in immunocompromised *mdx* mice has allowed for the selection and characterization of cyclin D2 (CCND2) as the lead candidate [131]. CCND2 is a member of the cyclin family of proteins responsible for cell proliferation and cell cycle regulation. Importantly, the study has revealed the presence of other functions of this gene in myogenesis and has demonstrated the feasibility of targeting genes, other than myostatin, capable of enhancing muscle regeneration. Although a promising candidate for the treatment of DMD using cell-mediated regenerative approaches, the function of CCND2 in SCs still remains to be elucidated and further testing will be required before treatments targeting CCND2 expression in muscle can be translated into clinical applications.

An additional high-content/HTS platform has been reported by Nierobisz *et al*. which utilized human primary cells for the identification of compounds that could improve muscle recovery (Table 1) [89]. This screen was performed using the Prestwick Chemical Library and a library consisting of 502 purified natural products (Enzo Life Sciences) and was designed to primarily identify and cluster compounds into specific categories thought to be important for muscle function such as cell survival, cell proliferation and myogenic lineage commitment. Among the compounds identified, Geraldol, a flavonoid that inhibits the activity of several enzymes including caspase-1, and Bromopride, a dopamine-antagonist, were shown to consistently and reproducibly induce significant increases in cell proliferation. Additional studies will be necessary before Geraldol and Bromopride can be assessed for their potential applications in DMD, but it can be envisioned that these compounds could be used to facilitate expansion of cells in culture and, therefore, may have applications in cell-mediated regenerative approaches to DMD which require large amounts of cells to be generated prior to transplantation into patients.

More recently, Saccone *et al*. elegantly demonstrated how HTS can be implemented in the discovery of novel molecular networks that influence muscle regeneration and muscle repair [90]. The authors used a combination of approaches which included gene expression microarray, genome-wide chromatin remodeling, small RNA sequencing (RNA-seq), and microRNA (miR) HTS to identify genes and miRs that influence the expression of specific paracrine factors that can either stimulate or inhibit regeneration mediated by SCs [90]. These miRs have now become novel targets to be pursued and further investigated for their ability to promote regeneration in DMD patients.

Finally, a fifth screen has been described by Xu *et al*. [91] and has utilized zebrafish blastomere cells to identify compounds that alter the development of skeletal muscle. Cells were isolated from a transgenic model expressing a dual reporter system capable of discriminating between muscle progenitors marked by the expression of GFP and differentiated muscle cells that instead, expressed a red fluorescent protein (mCherry). Of the 2,400 compounds screened, six were shown to increase both reporters, but only Forskolin, an activator of adenylate cyclase previously shown to play an active role in myogenesis [138,139,142,143,144,145,146], was able to increase proliferation of mouse muscle stem cells *in vitro.* Interestingly, Forskolin did not have any beneficial effects following transplantation of SCs expanded *in vitro* for five days in the presence of the compound and then transplanted into TA muscle of *mdx* mice. Better effects were achieved when Forskolin was used in combination with two other activators of myogenesis, basic fibroblast growth factor (bFGF) and the small molecule 6-bromoindirubin-30-oxime (BIO) a known inhibitor of glycogen synthase kinase 3 beta (GSK3β). The use of this cocktail in iPSCs and differentiating Embryoid Bodies (EB) exposed for seven days to the chemical mixture was shown to promote myogenic specification of iPS [91]. These results are encouraging because they demonstrate the possibility of using HTS to identify factors that influence differentiation of iPS cells further expanding the applicability of this technology to the treatment of DMD.

## 7. HTS of Compounds for DMD Using Zebrafish Models

Zebrafish models are becoming powerful tools in HTS due primarily to the possibility of utilizing these organisms to perform functional *in vivo* screens combined with a number of technical and practical reasons, as described and reviewed extensively elsewhere [147,148,149,150]. The fact that many human disease-related genes have orthologues in the zebrafish make these models particularly appealing systems to study biological process and for performing drug screening [149,151,152,153,154]. Moreover, there are multiple models of zebrafish that have been generated to study disease systems and, when those are not available, efficient gene expression knock out during early stages of development can be obtained through RNA interference using morpholino oligonucleotides. In addition, their ability to develop within eggs allows for their growth in 96-well plates to which compounds can be added at the necessary concentration for testing without excessive wasting of material and, therefore, allows the platform to remain cost effective. Finally, the transparency of zebrafish embryos permits studying the effects of compounds using optical systems, providing an ideal method to follow their development and identify compounds that are therapeutically relevant.

In the neuromuscular field, zebrafish models have proven to be a valuable system to study molecular mechanisms involved in human skeletal muscular dystrophy, dilated cardiomyopathy and hypertrophic cardiomyopathy [155]. Skeletal muscle represents the largest organ, even during early stages of development and can easily be identified in live animals, rendering this model particularly suitable for conducting HTS. Defects in muscle function and muscle contraction are often associated with defects in motor function providing an easy and convenient outcome measure for the identification of active compounds or other biologically active molecules using gain- or loss-of-function studies. Finally, dystrophin and DAPC are highly conserved in zebrafish and ablation of dystrophin expression leads to sever defects in motor function that can be visualized as early as day 2 post-fertilization and usually results in death within the first two weeks of life [156]. To date, at least two zebrafish mutants carrying defects in the dystrophin gene have been identified and characterized [156,157,158,159,160,161,162]. The *sapje* carries a nonsense mutation in exon 4 of dystrophin [157] while the *sapje-like* mutant has been shown to contain a mutation within the dystrophin exon 62 donor splice junction causing a frame shift in the mRNA coding reading frame and premature arrest of protein synthesis [163].

The *sapje* and *sapje*-like models have recently been used in two HTS aimed at identifying FDA-approved drugs that could be used to preserve muscle integrity. The study employed a two-step system in which the primary screen was performed using a series of eight compounds pooled into each of the wells assayed, while subsequent secondary screens were used to narrow down the primary hits and to select compounds that were active among those present in the pool of molecules initially tested. Of those, Aminophylline, a phosphodiesterase inhibitor known to increase the levels of intracellular cAMP, and Sinedafil, a drug known to inhibit the enzyme phosphodiesterase-5 (PDE5), were able to extend survival and to restore muscle integrity [86,87,88]. Although more work will be required before this drug can be tested in the clinic and before the mechanisms of action of this drug on muscle can be fully understood, this proof-of-concept study has demonstrated the utility of the zebrafish model in HTS and the feasibility of identifying drugs that could preserve muscle integrity using a live organism.

However, the inability of the zebrafish model used to faithfully recapitulate and predict effects in mammalian systems observed in the study reported by Xu *et al*. [91] also raises questions on the feasibility of using this organism as the only source of hits’ selection for preclinical and clinical stages of development of lead candidates. As a result, secondary and tertiary screens in additional organisms, such as mouse models or other mammalian species more closely related to humans, may be required before any claim can be made on the therapeutic relevance of active molecules identified using zebrafish models.

## 8. Conclusions

To date, less than 1500 compounds have been approved for use in humans and are available on the market. Among those, the majority were identified more than 20 years ago. This is in part due to the fact that it takes about 15 years for a drug to move into the commercialization pipeline from its first discovery to the late stages of marketing. Notably, the costs associated with the Research & Development (R&D) process can reach values of billions of dollars when performed in the industry sector. These costs reflect not only those associated with the actual expense of completing all stages required to get approval from the FDA or other regulatory agencies outside the US, but also those required to absorb the costs incurred by the many other drugs that, within the same company, fail during early or late stages of drug development. Furthermore, the tedious task of identifying new drugs in combination with a slow economy has forced many of the pharmaceutical companies to focus their research efforts on a limited number of targets or, as witnessed in the last few years, at limiting their drug discovery platforms only to drugs that are already approved and that could be refurbished for new applications further reducing the number of new molecules that are brought into market.

Under this scenario, it is not surprising that the US National Institute of Health (NIH) under the direction of Francis Collins has begun to promote new initiatives that could move promising therapeutic applications into the R&D pipeline [164]. New funding mechanisms are now available to academic investigators capable of supporting early stage drug discoveries and medicinal chemistry as well as preclinical and clinical studies which can now be conducted in academic institutions or other facilities funded by the NIH. This new infrastructure will be able to support a wide range of applications including the development of new therapies for the treatment of rare and neglected diseases otherwise dismissed by most companies due to modest market sizes. Similar initiatives are expected to begin soon from other international and government agencies around the world.

Although HTS has only recently been implemented in academia, the number of screenings that have been performed and assays that have been optimized thus far are impressive. The results obtained to date suggest that this technology can have a profound impact in the development of therapies to treat many disorders and Duchenne in particular. As academic investigators that have access to HTS facilities from major universities become familiar with the technology, assays are likely to become more refined, very sensitive and highly specific. A factor that is likely to be a major contributor to the success and expansion of HTS facilities in academia is the possibility of utilizing the knowledge of experts in the field who have spent the vast majority of their career on studying the same disease and even a specific aspect of the disease and that, therefore, have a wealth of knowledge superior to that often seen in the industry. In addition, the costs of optimizing and perfecting a specific HTS assay are generally significantly lower when the screening is performed in an academic institution compared to those normally encountered by industries. This is primarily due to the presence of infrastructures that are supported by academic institutions through state and government funds and that allow investigators to have access to those facilities at minimal costs. Among the downsides of optimizing HTS screening platforms in an academic setting is often the time required to develop the assay due to limitations on the number of academic personnel that can be dedicated to the project. Nonetheless, once optimized, assays can become powerful platforms that can be used in larger pharmaceutical settings to screen large libraries and compound repositories. As a result, academia is becoming an ideal ally for the industry. Close collaborations between companies and academia throughout different stages of drug development will become a critical component for the successful development of new therapies for DMD and other disorders. Finally, the generation of repositories containing compounds that have previously been optimized by pharmaceutical companies based on their drug-like properties, but that were unable to make it into the market due to lack of efficacy in clinical studies, is likely to accelerate the number of new drugs that can be moved into the market in a more efficient and cost-effective manner. Government agencies like the NIH will ultimately play a key role in establishing new relationships between the public and private sectors to ensure that the intellectual properties of new drugs and their applications for diseases, other than those they are initially intended for, are adequately protected.
